# The impact of COVID-19 pandemic on medical students’ mental health and sleep quality in Jordan: a nationwide cross-sectional study

**DOI:** 10.1186/s43045-021-00150-4

**Published:** 2021-10-26

**Authors:** Adnan Raed Alnaser, Rayan M. Joudeh, Osama A. Zitoun, Abdelkader Battah, Israa Al-Odat, Mohammad Jum’ah, Arwa A. Battah

**Affiliations:** 1 College of Medicine, Sulaiman Al Rajhi University, P.O. Box 777, Al-Bukayriyah, Al-Qassim 51941 Saudi Arabia; 2 School of Medicine, The University of Jordan, Amman, 11942 Jordan; 3 Faculty of Medicine, Al-Balqa Applied University, As-Salt, Jordan; 4 Saint Michael’s Medical Center, New York Medical College, Newark, NJ USA

**Keywords:** COVID-19 pandemic, Psychological distress, Mental health, Sleep quality, Medical students

## Abstract

**Background:**

COVID-19 pandemic is expected to affect the mental health, especially among medical students. Data from the literature in Jordan are scarce, especially during the second wave of the pandemic. We aimed to assess medical students’ level of fear, prevalence of depressive and anxiety symptoms—represented in psychological distress (PD)—and sleep quality (SQ) amid the current pandemic of COVID-19. A total of 2104 students were included through convenient sampling from the six schools of Medicine in Jordan. Online-based questionnaire using Patient Health Questionnaire-4 (PHQ-4) scale, Fear of COVID-19 scale (FCV-19S), and Sleep Quality Scale (SQS) was used to collect the data. Chi-square, *t*-tests, and ANOVA were used to establish the associations.

**Results:**

88.4% and 47.4% of the students were found to have PD and poor or just fair sleep quality on SQS, respectively, with PD ranging from mild (18.6%) to severe (42.1%). Calculated FCV-19S score was 14.62 (SD=5.38), indicating high level of fear. Students with excellent SQ had significantly lower rates of depression, anxiety, and PD as compared to those with good, fair, and poor SQ (*P* < 0.001 for all).

**Conclusion:**

Jordanian medical students appear to be especially susceptible to COVID-19 pandemic impact on mental health and reported high rates of PD. While rates of COVID-19 fear are still considered high, they are remarkably lower than that reported in early studies. We strongly recommend providing resources and access to professional mental health care to students reporting poor SQ and/or symptoms of anxiety and depression.

**Limitations:**

Using a cross-sectional design, online-based survey, convenient sampling, and scarcity of local literature are among the inevitable limitations caused by the pandemic that have prevented us from drawing cause-effect associations.

## Background

At the end of 2019, the SARS-CoV-2 (COVID-19) virus was detected in Wuhan, China. With the international exchange and travel, the virus was spread globally, resulting in an ongoing pandemic [1]. In March 2020, the World Health Organization declared the outbreak of the novel coronavirus (COVID-19) as a pandemic [2]. Relatively, COVID-19 was first detected in Jordan in March 2020; in order to prevent COVID-19 propagation in Jordan, the government enforced martial law which led to a national curfew on March 21, 2020, and this was followed by a declaration of a state of a health emergency. According to the data from the Ministry of Health, Jordan faced two waves of COVID-19. The first wave reached the peak in mid-November 2020, at which the total number of confirmed cases reached 174,335 and the death toll reached 2116. The second wave reached the peak at the end of March 2021 with a total number of confirmed cases of 605,007 and a death toll exceeding 6700 (https://corona.moh.gov.jo/en). In both waves, all educational institutions including medical schools in Jordan were closed and all forms of face-to-face teaching and training were suspended and converted into online education [3].

A medical student (MS) is known to experience high-stress levels that can disturb his or her physical and mental health, which in many cases can progress to cause anxiety (AX) and/ or depression (Dep) [4]. The worldwide prevalence rate of AX and Dep among MSs is 33.8% and 27.2%, respectively, with the highest prevalence of AX reported in the Middle East and Asia [5, 6]. Several factors can influence the psychological state such as sex, personality traits, believes, and socioeconomic status [7]. However, previous research has found that MSs are subject to some unique academic and non-academic stressors that might predispose them to psychological distress (PD). Academic stressors include having problems in understanding the syllabus, problems with reading from new extensive textbooks, long studying hours, irregular schedules, high study load, frequent exams, high sense of competition with others, and doubts about academic performance. Non- academic stressors include financial stress, high familial expectations, and fear of future failure in the medical career [4].

COVID-19 pandemic circumstances are expected to predispose to significant PD with variable degrees of fear, AX, and Dep, especially among those who are quarantined. A recent study from the USA reported that the prevalence of depressive symptoms was 3-fold higher during the COVID-19 pandemic when compared with the period prior to the pandemic [8]. Notably, MSs who experience depressive symptoms have an increased risk of dropping out of medical school [9], feeling less confident, and even having increased thoughts of harming their future patients [10] in what can be named as “empathy fatigue” [11].

Fear of the COVID-19 pandemic is a global issue and causes significant mental health problems, especially among MSs due to the nature of their lifestyle [12]. The experience of online learning adds to the existent stress, especially as MSs’ self-confidence decreases amid fears of lacking the essential clinical skills that are usually gained during hospital training [3]. In addition, the stress is exacerbated by fears of being at more risk of getting infected with COVID-19 or spreading the infection to their beloved ones, and not to forget the distress accompanied by being trained to see patients suffering from COVID-19 infection or even die due to its complications [13]. Continuous feelings of fear result in deterioration of study and work performances [14]. Due to the increased fears and concerns, a new kind of discrimination and stigmatization towards people infected with COVID-19 has emerged, and this has even further increased the fear of COVID-19 [15].

Sleep is an important biological and behavioral part of human life as it represents averagely one-third of our life span [16]. Sleep and mental health have a bidirectional relationship, as sleep problems can be a symptom of mental health problems [17] and predispose to psychological illnesses like AX and Dep [18]. Sleep disorders are common among MSs due to heavy study load together with other stressors, and these disorders are associated with poor academic achievement [19–21].

To the best of our knowledge, no studies were done in Jordan assessing the fear of COVID-19, the level of PD, and sleep quality during the second wave of COVID-19 pandemic. Therefore, we aimed in this study to assess the current situation of Jordanian MSs’ level of fear, the prevalence of depressive and AX symptoms, sleep quality, and the association of the fear with PD and sleep quality. We also aim to compare the current levels of PD, fear, and sleep quality with prior levels reported by several studies at the beginning of the COVID-19 pandemic and even studies from the pre-pandemic period in order to acquire a broad perspective of the pandemic’s impact on mental health.

## Methods

### Study setting and participants’ recruitment

We used a cross-sectional descriptive design to conduct the study. Our target population was MSs and interns from all levels (i.e., first-year to internship year) across the six medical schools in Jordan: The University of Jordan, Mut’ah University, Jordan University of Science & Technology, Hashemite University, Yarmouk University, and Al-Balqa Applied University. The estimated number of all MSs in Jordan is around 10,000. A respondent-driven sampling approach was adopted to recruit the participants. We contacted the dean of each of the six medical schools in Jordan and gained their permission to distribute the questionnaire to the students. After getting permission from all schools of medicine, we reached the student representatives for each batch across the universities to facilitate distributing the questionnaire for eligible participants. The questionnaire was sent to all students enrolled in the medical program through emails and was posted in the main official social media groups of each batch (e.g., Facebook, WhatsApp, Linked In, and Twitter). These groups are private groups, including MSs only, and under the supervision of the student’s representative of the concerned batch. We were able to reach responses from a total of 2104 participants in our study during the period between 21 and 30 April 2021. This period was during the COVID-19 second wave in Jordan during which the reported cases were close to 7000 and the death toll was exceeding 100 per day according to official figures from the Ministry of Health [22].

### Questionnaire administration

The students were invited to participate in the study with a cover letter briefly introducing the study protocol, aims, and anonymity of the data. The questionnaire was administered in the form of an Online-based Google Form survey link. Students were asked to fill the questionnaire after acknowledging an informed consent. It was clearly stated that participation is voluntary, and the participant can withdraw at any point in time without any consequences. It was also clarified that no direct benefits will be gained from participation aside from contributing to an activity that will add to existing knowledge. No personal identifiers were obtained, and participants were encouraged to disclose the required information with transparency to help reach accurate conclusions.

### Measurement tool

A structured online-based English self-administered questionnaire was used for data collection. It consisted of four parts: the participants’ demographics section, the Patient Health Questionnaire 4 (PHQ-4 scale), the Fear of Coronavirus-19 Scale (FCV-19S), and the single item Sleep Quality Scale (SQS).

#### Demographics

Participants’ demographic characteristics were collected, including age, gender, university, study level (first year to internship year), and planned future specialty, which was categorized into medical specialities, surgical specialities, psychiatry, and no plans to specialize. Also, personal and family/close friend history of mental illness was assessed using direct Yes/No questions: do you have any mental illness? and does any of your family members/close friends suffer from any mental illness?

#### Psychological distress (PHQ-4 scale)

Psychological distress was assessed using the validated Patient Health Questionnaire 4 (PHQ-4 scale) [23]. The PHQ-4 consists of four items answered on a 4-point Likert-type scale: not at all, several days, more than half days, and nearly every day. It allows ultra-brief and accurate measurement of core features of Dep and AX by combining the two-item measure (PHQ-2), consisting of core criteria for Dep, and a two-item measure for AX (GAD-2), both of which have independently been shown to be good brief screening tools. The PHQ-4 scale is a reliable and valid tool for screening for Dep and AX among college students as proved by psychometric testing with a reported *α*= 0.81 [24] and *α*= 0.73 in our study. The total score of PHQ-4 ranges between 0 and 12 and is represented as the following PD categories: none (0–2), mild (3–5), moderate (6–8), and severe (9–12). Previous research has established that a score of 3 or greater on the Dep subscale or AX subscale represents a reasonable cut-off point for identifying potential cases of Dep or AX, respectively [25]. Therefore, these cut-offs were used to label the participants in our study as positive screening as yes and negative screening as no.

#### Fear of COVID-19 (FCV-19S)

Fear of COVID-19 was assessed using the Fear of Coronavirus-19 Scale (FCV-19S) that was developed and validated by Ahorsu et al. [26]. FCV-19S composed of seven items answered on a 5-point Likert-type scale (strongly disagree, disagree, neutral, agree, strongly agree). The minimum possible score for each item is 1 (strongly disagree), and the maximum score is 5 (strongly agree). The total score is calculated by adding up the items’ scores (range between 7 and 35). The higher the reported score on FCV-19S, the higher the fear of the COVID-19 pandemic. Since FCV-19S was developed back in March 2020, it has been validated on several populations including college students [27].

#### Sleep quality (SQS)

To assess participant’s SQ, we used the validated single-item SQ Scale (SQS) [28]. SQS is a self-rated, global SQ assessment tool developed based on a literature review of key aspects of SQ, critical components of the Pittsburgh SQ Index (PSQI), the morning questionnaire-insomnia (MQI), and direct expert and patient input. Participants are asked to rate their SQ in the past 7 days based on the following factors: total sleeping hours they got each night, how easily they fell asleep, how often they woke up during the night (except to go to the bathroom), how often they woke up earlier than they had to in the morning, and how refreshing was their sleep. The rating is on a 1 to 10 scale, with 1 being terrible and 10 excellent. A score of ≤3 is regarded as poor, 4 to 6 regarded as fair, 7 to 9 regarded as good, and 10 regarded as excellent SQ.

### Ethical considerations

The study protocol was written according to the ethical principles of the declaration of Helsinki, and it was reviewed and approved by the Institutional Review Board (IRB) at University of Jordan, Amman, the Hashemite Kingdom of Jordan on 20/04/2021 (reference number: 101/2021/9341) in meeting No. 7/2021.Written informed consent was obtained from all participants before filling the questionnaire. The data was collected and proceeded confidentially and stored eventually on a personal computer device that only the authors have access to.

### Statistical analysis

After finalizing data collection, data was extracted from Type-form into a customized Microsoft Excel (2016) password-protected spreadsheet then imported into IBM SPSS Statistics for Windows, version 25 (IBM Corp., Armonk, NY, USA) for analysis. Responses were first examined for missing values, outliers, and assumptions of linearity, normality, and homoscedasticity. Descriptive statistics were generated to summarize the quantitative and categorical variables. The summary scores of the PHQ-4 scale, FCV-19S, and SQS were calculated and categorized accordingly. PHQ-4 total score was categorized into normal, mild, moderate, and severe. Anxiety (AX) and Depression (Dep) subscales of PHQ-4 were categorized into positive screening/negative screening. SQS score was categorized into poor, fair, good, and excellent. The reliability of the PHQ-4 scale items and FCV-19S items was assessed using Cronbach’s alpha to confirm the internal consistency of each scale. Appropriate Chi-square, t-tests, and ANOVA were used to establish the associations between the covariates and AX subscale, Dep subscale, PHQ-4 total score, and FCV-19S summary score. Bonferroni correction was used to adjust the multiple comparisons for ANOVA associations. Pearson correlation coefficient was used to determine the relationships between FCV-19S, PHQ-4 total score, AX sub-score, and Dep sub-score. A *p*-value of *α* < 0.05 was set to determine the statistical significance of the reported results.

## Results

### Demographic characteristics

A total of 2104 MSs from the six medical schools were included in our study: Al-Balqa Applied University (23.5%), the University of Jordan (22.6%), Mutah University (21.5%), the Hashemite University (14.1%), Yarmouk University (10.8%), and Jordan University for Science & Technology (7.5%). Their background characteristics are demonstrated in Table 1. Females constitute more than half the sample (58.7%) and the mean age of all participants was 21.3 (range 17–28 years). Nearly one-quarter (27%) of the MSs declare having a personal mental illness (not specified), while 50.9% declare having one or more family members/close friends who had a mental illness (not specified). Almost half of the MSs were found to have poor or just fair SQ on SQS (47.4%).

**Table 1:** Demographic characteristics of medical students in Jordan and its association with anxiety, depression, PHQ-4 total score, and FCV-19S score (*n*= 2104)

Variables	Anxiety subscale screening			Depression subscale screening			PHQ-4 total score		FCV-19S score	
	***Positive (n=1488)***	***Negative (n=616)***	***p-value***	***Positive (n=1475)***	***Negative (n=629)***	***p-value***	***Mean (SD)***	***p-value***	***Mean (SD)***	***p-value***
***Age*** (mean 21.30, SD 2.2)	21.31 (2.2)	21.23 (2.1)	0.47	21.30 (2.2)	21.24 (2.1)	0.60	NA		NA	
***Gender:***										
Female (*n*= 1234)	875	359	0.82	856	378	0.38	7.05 (3.7)	0.59	14.59 (5.3)	0.75
Male (*n*= 870)	613	257		619	251		6.96 (3.6)		14.67 (5.5)	
***Study level:***										
1st year (*n*= 324)	230	94	0.054	221	103	0.44	6.89 (3.7)	0.19	14.65 (5.4)	0.18
2nd year (*n*= 481)	349	132		338	143		7.03 (3.5)		14.95 (5.6)	
3rd year (*n*= 298)	206	92		207	91		6.85 (3.8)		14.60 (5.4)	
4th year (*n*= 245)	156	89		169	76		6.70 (3.9)		13.94 (5.1)	
5th year (*n*= 294)	200	94		200	94		6.95 (3.6)		14.86 (5.5)	
6th year (*n*= 271)	204	67		206	65		7.49 (3.6)		14.18 (5.3)	
Internship (*n*= 191)	143	48		134	57		7.28 (3.4)		14.96 (5.0)	
***Personal history of mental illness:***										
No (*n*= 1536)	1091	445	0.61	1093	443	0.08	7.06 (3.6)	0.39	14.73 (5.4)	0.16
Yes (*n*= 568)	397	171		382	186		6.90 (3.7)		14.35 (5.3)	
***Family/close friend history of mental illness:***										
No (*n*= 1033)	736	297	0.60	725	308	0.94	7.03 (3.6)	0.87	14.73 (5.3)	0.40
Yes (*n*= 1071)	752	319		750	321		7.00 (3.6)		14.53 (5.5)	
***Sleep quality:***										
Excellent (*n*= 196)	101	95	<0.001	94	102	<0.001	4.83 (4.2)	<0.001	12.95 (5.0)	<0.001
Good (*n*= 910)	664	246		643	267		6.93 (3.8)		14.26 (4.7)	
Fair (*n*= 694)	525	169		538	156		7.75 (3.3)		15.37 (5.8)	
Poor (*n*= 304)	198	109		200	104		7.00 (3.0)		15.10 (6.1)	

### Psychological distress, anxiety, and depression

**Fig. 1:**
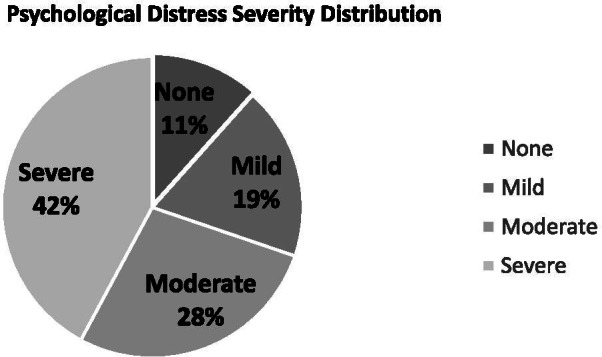
Psychological distress severity on PHQ-4 scale among medical students in Jordan (*n*= 2104)

Students’ responses to PHQ-4 items are summarized in Table 3. The calculated mean PHQ-4 total score was 7.01 (SD= 3.64), and Cronbach’s alpha= 0.73, confirming the scale’s internal consistency. A high proportion of the MSs was found to have PD (88.4%) ranging from mild (18.6%) to severe (42.1%) as illustrated in Fig. 1. Furthermore, more than two-thirds of the MSs screened positive for AX (70.7%), with a similar proportion screened positive for Dep (70.1%).

Sleep quality was significantly associated with AX screening, Dep screening, and PHQ-4 mean total score (*P* < 0.001 for all), as shown in Table 1. ANOVA with Bonferroni adjustment for the multiple comparisons of SQ association with AX, Dep, and PHQ-4 total score is demonstrated in Table 2. Students with excellent SQ had significantly lower rates for AX compared to those with good, fair, and poor SQ (51.5% Vs 73%, 75.5%, and 65.1%, respectively; *P* < 0.001, *P* <0.001, and *P* =0.006, respectively). Similarly, students with excellent SQ had significantly lower rates of Dep as compared to those with good, fair, and poor SQ (48% Vs 70.7%, 77.5%, and 65.8%, respectively; *P* < 0.001 for all). Furthermore, excellent SQ was associated with significantly lower PD (i.e., lower PHQ-4 total scores) as compared to good, fair, and poor SQ (4.83 Vs 6.93, 7.75, and 7.00, respectively, *P* < 0.001 for all) (Table 3).

**Table 2:** Bonferroni adjustment for ANOVA multiple comparisons of sleep quality with anxiety, depression, PHQ-4 total score, and FCV-19S score (*n*= 2104)

	***Anxiety screening***	***Depression screening***	***Total PHQ-4 score***	***FCV-19S total score***
**Sleep quality**	***F-value***			
	16.97	22.99	34.77	13.10
	***P-value***			
***Poor < fair***	0.004	0.001	0.014	1.00
***Poor < good***	0.052	0.62	1.00	NA
***Good > poor***	NA	NA	NA	0.13
***Excellent < poor***	0.006	< 0.001	< 0.001	< 0.001
***Good < fair***	1.00	0.15	< 0.001	< 0.001
***Excellent < fair***	< 0.001	< 0.001	< 0.001	< 0.001
***Excellent < good***	< 0.001	< 0.001	< 0.001	0.01

**Table 3:** Participants’ responses to PHQ-4 items (*n*= 2104)

PHQ4 items	***Not at all***	***Nearly every day***	***More than half days***	***Several days***
***Feeling nervous, anxious or on edge***	414 (19.7%)	368 (17.5%)	375 (17.8%)	947 (45.0%)
***Not being able to stop or control worrying***	690 (32.8%)	243 (11.5%)	324 (15.4%)	847 (40.3%)
***Feeling down, depressed or hopeless***	434 (20.6%)	353 (16.8%)	397 (18.9%)	920 (43.7%)
***Little interest or pleasure in doing things***	556 (26.4%)	385 (18.3%)	414 (19.7%)	749 (35.6%)

### Fear of COVID-19

The combined mean score for the FCV-19S was 14.62 (SD= 5.38) which exceeded the midpoint for the total score range (= 14), indicating an elevated level of fear of the COVID-19 pandemic among the participants. The Cronbach’s alpha of FCV-19S was 0.86, which confirms the scale’s internal consistency. Sleep quality was found to be significantly associated with the FCV-19S score (*P* < 0.001), as indicated in Table 1. MSs with excellent SQ scored significantly lower FCV-19 scores when compared to those with good, fair, and poor SQ as shown in Table 2 (12.95 Vs 14.26, 15.37, and 15.10, respectively, *P* < 0.001 for all).

### Association between fear of COVID-19 and psychological distress

Table 4 shows the relationship between FCV-19S scores and AX, Dep, and PHQ-4 total scores. It was also found that MSs who screened positive for AX or Dep also tend to have more fear of the COVID-19 pandemic on FCV-19S. The mean FCV-19S score for students who screened positive for AX is 14.88, while the mean score for those who screened negative is 14.00 (*P*= 0.001). Similarly, MSs who screened positive for Dep scored a mean score of 14.87 as compared to 14.05 in those who screened negative (*P*= 0.001). A weak but significant positive correlation was found between FCV-19S mean score and PHQ-4 total score (*r*= 0.13, *P* < 0.001).

**Table 4:** Association between Fear of COVID-19 on FCV-19S scale and (anxiety, depression, and PHQ4 total score) (*n*= 2104)

	Fear of COVID-19 (FCV-19S scale)			
	***Mean (SD)***	***P-value***	***Pearson correlation***	***P-value***
***Anxiety:***			NA	
Positive	14.88 (5.3)	0.001		
Negative	14.00 (5.5)			
***Depression:***				
Positive	14.87 (5.4)	0.001		
Negative	14.05 (5.3)			
***PHQ4 total score***	NA		0.13	< 0.001

### Association between anxiety and depression

Table 5 shows the relationship between AX and Dep subscales. We found that MSs who screened positive for Dep tend to screen positive for AX as well, and vice versa (*P* < 0.001). A markedly large proportion (84.5%) of MSs who screened positive for Dep had also screened positive for AX. Similarly, a close proportion (83.8%) of MSs who screened positive for AX also had the same for Dep. Consequently, a strong positive correlation was found between AX and Dep subscales (*r*= 0.504, *P* < 0.001).

**Table 5:** Association between anxiety and depression subscales using crosstabs and Pearson correlation (*n*= 2104)

	Chi-square			Sub-scores correlation			
	***Anxiety (Positive)***	***Anxiety (Negative)***	***P-value***	***Anxiety score (SD)***	***Depression score (SD)***	***Pearson correlation***	***P-value***
***Depression (positive)***	1247	228	< 0.001	3.51 (2.1)	3.50 (2.1)	0.504	< 0.001
***Depression (negative)***	241	388					

## Discussion

The ongoing COVID-19 pandemic has affected nearly all aspects of life. The impact on mental health is becoming an increasingly important aspect to consider, especially after the second wave of the pandemic. Saying that, MSs are among the populations known to experience high-stress levels caused by the nature of their studies, sleep disturbances due to the high workload, and continuous exposure to patients in distress [29] and are thus prone to be affected by the pandemic. Moreover, several negative psychological consequences can be triggered by social isolation and uncertainty about the future [30], conditions which are applicable to the long quarantine duration in the Kingdom of Jordan.

The current study assessed the level of PD and fear amid the COVID-19 pandemic and examined the association between PD, fear, and SQ. This study has surveyed 2104 MSs during the peak of the second wave of the COVID-19 pandemic in Jordan and covered students from all six schools of Medicine in the country. With over 2000 respondents, this survey covered a large and representative sample of the population of MSs in Jordan. Also, the survey was administered during the most critical phase of the second wave of the pandemic at times where the country was announcing approximately 7000 newly confirmed cases per day.

Jordanian MSs appear to display high levels of PD during the COVID-19 pandemic. In our survey, around 70% of students screened positive for AX and Dep, respectively. Similarly, 75% of Jordanian MSs surveyed during Spring 2020 using the Epidemiologic Studies-Dep Scale (CES-D) suffered from depressive symptoms [31]. In comparison, only 24.3% and 30.6% of US MSs who were surveyed during April 2020 screened positive for Dep and AX, respectively [32], and nearly half of MSs in UAE reported mild to severe levels of AX at the onset of the pandemic [33]. Moreover, the rates of AX and Dep in Jordanian MSs are higher than those in other Asian countries such as Bangladesh, where around 66% had different levels of AX and 50% had Dep symptoms during May 2020 [34]. The prevalence is also similar among Chinese college students, of which 56.8% reported Dep symptoms during March 2020 [35].

However, the high rates of Dep and AX symptoms among MSs in our survey could not be completely traced to the current COVID-19 pandemic. In a survey of 600 Jordanian college students done before the pandemic in 2018, students showed a moderate level of Dep and a severe level of AX as measured by the Dep, AX, and Stress Scale DASS-21 [36]. Back in 2017, another survey reported that almost 55.7% of students exhibited a variable degree of depressive symptoms [37], demonstrating the enduring prevalence of PD among college students in Jordan. These reports suggest that the high rates of Dep and AX symptoms among Jordanian MSs is actually a long-standing issue which got inflated from the added stress and influences imposed by the current pandemic.

As to SQ, 14.4% of respondents reported poor SQ on SQS, a prevalence that is lower than that reported by several studies at the beginning of the pandemic. Comparably, the prevalence of poor SQ in an earlier study done during spring 2020 on Jordanian MSs, which assessed SQ using Pittsburgh’s Sleep Quality Index (PSQI), was 76% [31]. Also, an Italian study of 6519 adults during the lockdown at the beginning of the pandemic have assessed SQ using Medical Outcomes Study–Sleep Scale (MOS-SS) and concluded that around 55% of respondents had poor SQ [38], and another study from China reported that about 36% of the general population were poor sleepers during the beginning of the COVID-19 pandemic [39].

Fear is also expected to be experienced during the current COVID-19 pandemic consequences [40]. The mean score on the FCV-19S was 14.62 (SD= 5.38), exceeding the midpoint for the total score range (= 14) to indicate a high level of fear of the COVID-19 among the participants. Nevertheless, the mean score in our study is relatively lower than several studies that assessed fear at the onset of the pandemic. Surveys of 5423 MSs in Vietnam [41], 606 university students in Spain [42], 433 university students in UAE [33], and 228 university students in Russia and Belarus [43] during April and May 2020 reported a mean score of 16.7 (SD= 5.3), 16.79 (SD = 6.04), 16.6 (SD= 6.3), and 18.0 (SD= 4.5), respectively, on FCV-19S. As our survey was administered during the second wave after more than 1 year from the onset of the pandemic, the slightly lower rates of fear and poor sleep in our study as compared to early studies can be explained by adaptation, availability of the vaccines, and a better understanding of the situation with more facts and fewer rumors promoted.

The high alarming rates of PD obviously demonstrate the persistent impact of the COVID-19 pandemic. Prolonged social distancing and quarantine that precipitated further isolation may have an ongoing effect on the students, ultimately leading to PD and fear, which did not significantly differ between junior and senior students. In contrast, a recent study from Spain has shown that first-year students reported high significant levels of fear of COVID-19 when compared with senior students [42]. Although several studies have shown higher rates of PD among females than males [31, 32] our study has not found significant gender differences in relation to Dep, AX, or poor SQ rates. Also, the personal history and experience of mental illness affected neither the rates of PD nor the fear of COVID-19.

Poor SQ is a known risk factor for the development and progression of several psychiatric diseases including AX and Dep [44, 45]. Consistently, a significant correlation was found in our survey between PHQ-4, FCV-19S, and SQS scores. Expectedly, participants who screened positive for Dep and/or AX had higher levels of fear of the COVID-19. Respondents with excellent SQ showed significantly lower rates for AX and Dep as compared to those with good, fair, and poor SQ, respectively. Hence, as non-pharmacological sleep interventions, such as relaxation training, sleep restriction therapy, and biofeedback, were found to be effective in reducing the severity of depressive symptoms [46], they would be strongly recommended to participants reporting poor SQ. Also, students screening positive for depression and/or anxiety would be advised to seek counseling via professional mental health services including telepsychiatry resources. Furthermore, multidisciplinary collaborative action should still be taken to manage the PD and poor quality of sleep during this uncertain time amid the COVID-19 pandemic [38].

Over a year and a half have passed since the pandemic took hold and hit the globe resulting in persistent disturbance across all levels of life. Fortunately, the COVID-19 vaccines were developed and approved by World Health Organization (WHO) for emergency use in late 2020 [47], and the first became available in Jordan in January 2021 [48]. In addition, the health care infrastructure in Jordan improved significantly since the onset of the pandemic. With the availability of vaccines and improving health care facilities, the levels of fear and PD are expected to decrease when compared to the levels at the onset of the pandemic.

### Strengths and limitations

Up to our knowledge, this study has surveyed the largest sample of students from all schools of Medicine in Jordan, allowing for an evident element of representation and facilitating the extraction of comprehensive results. This is the first study to use the newly developed FCV-19S among MSs, which has been used in several countries to assess fear of COVID-19 at the onset of the pandemic, in Jordan, and gain an insight into the current situation as compared with early studies. However, several limitations persist. The cross-sectional design prevents us from drawing cause-and-effect relationships and confirming how mental health is affected by the spread of COVID-19. Also, although it is important to measure the subjective observation of distress and sleep, objective estimates of PD and sleep including actigraphy and sleep diary could be helpful to strengthen our findings. This in addition to using an online sample with convenience sampling are some inevitable limitations with the present COVID-19 restrictions. Another limitation is the deficiency of literature on PD and SQ among Jordanian MSs before the pandemic, making it harder to accurately assess the effect of the pandemic and associated quarantine on AX, Dep, and SQ. Thus, with the imminent resolution of the pandemic consequences, we would recommend implementing a similar survey on MSs outside the quarantine period to compare the levels of distress with the levels that were indicated during the pandemic.

## Conclusions

Jordanian MSs appear to be especially susceptible to the impact of the COVID-19 pandemic on mental health and reported high rates of positive screening for AX and Dep. The demanding and extensive nature of medical education and the persistent higher rates of PD among MSs as compared to the general population can place them as a vulnerable population to the effects of the COVID-19 pandemic and the resultant quarantine. While fear of COVID-19 and PD are still considered high, it is remarkably lower than that reported in early studies. These results will hopefully provide relevant information to guide policymakers and universities’ administrators in responding to the pandemic’s consequences on MSs’ mental health, and the authors would highly recommend supplying MSs with resources such as counseling and peer advocacy and suggesting professional mental health care to students who report poor SQ and/or symptoms of Dep and AX.

## Data Availability

Data are available upon reasonable request to the corresponding author.

## Abbreviations

MS: Medical student
AX: Anxiety
Dep: Depression
PD: Psychological distress
SQ: Sleep quality
SQS: Sleep Quality Scale
FCV-19S: Fear of Coronavirus-19 Scale
PHQ-4: Patient Health Questionnaire 4
